# Book Reviews

**Published:** 1817-12

**Authors:** 


### CRITICAL ANALYSIS

### *OF RECENT PUBLICATIONS,*

IN THE

### DIFFERENT BRANCHES OF PHYSIC, SURGERY, AND MEDICAL PHILOSOPHY.

*Surgical Observations; with a Quarterly Report of Cases in Surgery; treated in the Middlesex Hospital, in the Cancer Establishment, and in Private Practice: embracing an Account of the Anatomical and Pathological Researches in the School of Windmill-street.* By CHARLES BELL. 8vo. Longman and Co.

AFTER such a title, many readers will exclaim, "Quid dignum tanto feret hic promissor hiatu?" The following extract from the Preface will develope the author's design.

"The object of this work is to illustrate the principles of surgery by observations made in a public hospital and in a school of anatomy, where every thing is open to inspection, and where, consequently, the statements are made in the presence of many observers. The author does not intend to publish more than three volumes of cases. These he hopes will embrace the whole practice of surgery, and supply a book of reference for the history of surgical diseases, and the minute account of symptoms.

"This work was suggested by observing, that published cases contain only what is new and monstrous, and but few examples which may serve to initiate the young surgeon into the business of his profession. But, although the author began to take his cases with with the intention of illustrating the common matters of practice, he now hopes that they may sometimes have the interest of novelty also; since the close observation of what are called common cases has led to new views and improvements of practice, as well as to the illustrations of the acknowledged principles of the art. Every one must be convinced that there is room for a critical inquiry into the present state of surgery, and it cannot be more safely undertaken than in the form of Observations made at the bed-side of the patients.

"In published cases, a very common defect is too much consistency—matters proceed so smoothly, that when the young surgeon enters on the actual duties of his profession, he is troubled with adverse occurrences, for which he is quite unprepared: and he wonders to find his experience so different from what he has been led to expect from the perusal of cases. The utility of cases arises from the confessions of the surgeon which exercise the reader's mind, and enable him to anticipate the harassing difficulties of practice. Whoever proposes to publish useful cases, must have a full dependence on the candour and liberality of his readers, and forget those who lie in wait for occasions of rancorous criticism. He has to draw two parallel histories—the history of symptoms, and the history of his own mind, with his doubts and anxieties during the course of the disease. Just so far as the observer's mind is active, and the communication of his thoughts free, will the cases be useful.

"The author wishes to avoid that distortion which love of system produces in cases which are given in illustration of particular doctrines. He hopes to combine the interest and usefulness which arise from the perusal of cases, classed so as to enforce practical results, with the genuine and uncoloured statement which belongs to the records of an hospital.

"Those who feel interested to inquire, may find security for the fulfilment of this undertaking in a life hitherto given up to the improvement of the younger members of the profession—where the labour and the pastime have been only a variation in the manner, not in the object of pursuit,—namely, the improvement of anatomy, and its application. The author now enters on subjects of higher interest and greater magnitude, as relating to questions of life and death. In judging of his motive it will perhaps be recollected, that upwards of twenty years have been given to anatomy, and to the teaching of the acknowledged principles of surgery, without aiming at improvements in practice; that before he has entered critically on matters of practice, he has waited until half a life, spent in the laborious duties of teaching, together with the possession of the fullest opportunities, may be supposed to have matured his judgment.

"He is happy in thinking, that by this undertaking, he shall prolong the term of his connexion with his pupils, and continue to afford to them, though in the country or on service, the advantages of hospital practice and an extended experience, which at a distance from the capital are not easily obtained."

Twenty

Twenty years and half a life are sonorous words, but, like every thing in this world, they are only comparative. The youth fresh from the shop, views the man of forty as somewhat beyond his maturity, whilst the veteran is apt to consider him still in his boyhood. We are not now referring to Mr. Charles Bell's age, of which we know nothing; but, recollecting him as a young writer, and not over delicate in his remarks on his seniors, we acknowledge that we do not easily surmount our first impressions. This our readers will consider as a confession, and one which we wish them to keep in mind, should we express less satisfaction with individual parts of this work than we hope to do with the whole.

The first report on Cancer contains a short account of the institution of a cancer ward by the *first* Mr. Whitbread, which introduces the recommendation of the plan of compression by the *late* Mr. Whitbread.

"I have only (says Mr. Bell) to observe, that the essential part of this new plan of cure is the compression of the cancerous tumour, gently at first, and with a force gradually increased, till at last it is augmented to a very great degree: and that the means are these—if the cancer be open, the various holes and cavities are filled up from the bottom with chalk, finely levigated, and all the surface is thickly covered with hair powder; over this, long plaster straps are put, so as to cover the whole surface of the tumour, over this again are placed linen compresses, bound down with the turns of a roller, firmly applied, and of six yards in length; or over the first straps are laid a second set, bracing the parts more firmly than the first, over this a plate of lead, and lastly, the long roller is carried round the chest, compressing the whole."

A Report of the Medical Committee follows; by which it appears, that eight cancers in an ulcerated state, and eight in a scirrhous state, have been submitted to this treatment; that in some cases of open cancer considerable relief was afforded, but that the specific nature of the cancer remained the same, and, that in some instances, the fatal issue was hastened. In the scirrhous stage, the benefit was still more doubtful. We copy the following *penultimate* paragraph, as we may have hereafter occasion to refer to it again.

"Your committee, however, although they cannot lay claim to the discovery of a specific, have still the consolation to believe that they have in many cases succeeded in obtaining great alleviation of suffering; such alleviation as might, perhaps, induce some speculative minds, less disciplined by experience, to conclude, that they had at length succeeded in discovering a cure for cancer."

Some observations follow by the author. Here we expected a most minute description of those cases in which pressure had been useful, contrasted with those in which it had had proved useless, and had even done harm. From such sources of experience, and such habits of observation, we expected nothing less than instructions under what circumstances we might use pressure with safety or advantage, and such in which it would aggravate a most painful disease. If in this we expected too much, we might at least have been informed, that, after the most diligent research, the committee were not enabled to offer any instructions concerning the character of any local ulcers or tumours, in which they might expect benefit or injury from pressure. Another circumstance will probably strike the reader, "that the committee do not lay claim to a discovery of a specific." However, as far as the remedy goes, they have the consolation—of what?—of *adopting* a plan, the benefit of which, Mr. Whitbread witnessed under Mr. Young, who, though not named, we conceive is sneeringly alluded to as one of those "*speculating* minds, less disciplined by experience." Some remarks follow on the advantages of bandaging in sinuous ulcers, and of rest in some obstinate spreading sores. Reverting to the work recommended by Mr. Whitbread, we are told, the only successful case "is a cancerous lip;" and, in this, it is easy to prove, that THEY *are anticipated in the method of curing what is called cancer of the lip.*" What induced Mr. Charles Bell to commence this remark by *they*, we know not, but, when he concludes this sentence, by assuring us that rest and pressure will "infallibly cure malignant ulcers of the lip without excision;" we cannot help wishing he had taught us how to distinguish such an ulcer from a cancer, for we believe that many of the young gentlemen to whom he addresses himself, will recollect cancerous lip, for which the operation has been imperfectly performed, and in which, after stitching, long continued pressure, and rest, the disease has returned with such increased violence as to discourage all future attempts.

These events, however, show, that pressure may be useful in some cases which are not easily distinguished from cancer. This is the consolation, and by the passage above quoted, such would appear the implied "discovery of the committee," were it not for the penultimate paragraph of the "additional observations."

"And now (says Mr. Charles Bell,) it only remains for me to state, that the idea of destroying by pressure, dangerous tumours, which could not be extirpated with the knife, is familiar to me from my first entering upon the study of our profession. Mr. John Bell had an opinion, that it was possible to suffocate and subdue the action of vessels in tumours by the compress and bandage. This he was wont to illustrate by the effect of that bandaging of the limbs, by by which mendicants reduce the substance of their limbs to a third part of their natural bulk; by the example of the feet of the women of China, and other ingenious analogies. He argued, that, if the natural structure of the body could be moulded by pressure, why should not these formidable tumours?"

In these remarks and extracts we have omitted to notice some theories which we hope will be hereafter explained. According to the author's observation, pressure "does not subdue specific action. Nor is it desireable; that absorption of the matter of cancer should take place. In true cancer, there is a peculiar matter unlike the original structure, produced by the specific action, and deposited in the texture of the tumour. Now were it possible, that the compress and bandages did actually excite the lymphatics to absorb the cancerous disposition,"—we stop here, as we are afterwards told, "this a mere matter of opinion and speculation." Still it should be intelligible; matter and structure are two things, and ought not to be compared together. Still more difficult is it to conceive, how the lymphatics should *absorb a disposition.*

The second report on diseases and wounds of the larynx, and of the operation of bronchotomy, contains many just suggestions, but nothing new to those who have perused some of our numbers of the last and preceding volume. We wish Mr. Bell had been more explicit in the following short paragraph.

"In these four examples (says he, alluding to four preparations in the Museum) of disease of the larynx, we see the nature of the membrane of CROUP. It is formed by inflammation of the membrane lining the larynx and trachea, by which a proportion of coagulable lymph [more or less, according to the violence of the inflammatory action] is added to the mucous secretion. Accordingly, it assumes, in one instance, [No. 1], the appearance of concreted mucus; in another, [No. 4], the character of coagulable lymph."

Every one knows how uncertain a sense the sight is, and particularly when applied to wet preparations, seen through two or three mediums. Though, therefore, the youths (Mr. C. B.'s hearers) might seem ready at taking their master's word, and giving him credit for his accurate discrimination between coagulated lymph and concreted mucus, yet a teacher should always remember, that the apparent acquiescence or dutiful silence of a scholar, is no proof that he is satisfied with, or even that he fully comprehends his teacher.

The third report of diseased pharynx and œsophagus has some interesting and useful cases, with good practical remarks. The fourth, containing cases of fistula in perineo, is full

2

full of histories of those complicated evils with which the urinary, genital, and neighbouring parts are so often afflicted.

The fifth report is on fracture and dislocation of the spine, with injury of the spinal marrow; cases of fracture of the ribs, attended with emphysena and with caries. We copy the first case as a specimen of the mode in which young gentlemen should be admonished in lecture, and afterwards in print.

"*I am happy to meet you again, and especially, because there is a subject on which I wish to address you: and you must excuse me for saying, that it is a subject of which you are criminally negligent. It is easy to know whether or not a student be properly educated, by observing the things to which he attends in an hospital, just as you may know a gentleman of liberal pursuits by his conversation, and the objects which interest him. You will presently observe the application of this remark.

"There was an old Irishman, one of my patients in the accident-ward, when I left town, in whom I took much interest, and often I drew your attention to the case, and made you feel his sides. To many of you I explained his critical situation. Shall I confess I was concerned to observe the little attention that you paid to this subject?

"He was a man of sixty-five years of age. He had fallen from a ladder and struck his left side upon the corner of a chair; he remained at home for three days, until his master, having called on me, procured his admission here, to which you know his misfortune gave him a title without my influence. I found him sitting up in bed, suffering much from pain in his side, aggravated by a short cough. On examining the side, it was not possible to feel the ribs, but you might perceive other evidence of his ribs being broken in the emphysematous tumour which covered them. He was fat, with that looseness of skin which is characteristic of his years; the skin was blown up, forming a tumour extending from the ilium to the clavicle.

"There was no doubt that the rib was broken, and the lungs torn. I witnessed his situation with considerable uneasiness; but, as he could lie down, and as he repeatedly affirmed he was easy, but for the troublesome cough which he said gave him pain in his side, I was satisfied with ordering him to be bled, and to have a linctus for his cough. I sent to inquire for him in the afternoon: I visited him in the evening: I sent again in the morning: and I saw him at twelve o'clock. The tumour spread further over the breast, and over the hips; but nothing untoward occurred. He continued better the third day; on the fourth, he was still better. On the sixth and seventh day from the accident, the emphysema began to dissipate, and, by the common attention to confine the motion of the rib, and keep the circulation low, he quite recovered.

"* From Clinical Lectures."

"Here was a case in which more was present to the understanding, than visible to the eye; he who had never studied would pass it with indifference, but the pupil who had read what Dr. Hunter had written on this subject; what Portal had delivered to the Academy of Surgery; what experiments Hewson had made; or who had felt the interest which Mr. John Bell had given to this subject in his book of wounds; such a pupil would have looked with intense interest on the case. I wish that some among you had so attended to the case, I should not then have had to record the following instances."

Some very interesting cases follow, well worth recording, of fractured ribs, with emphysema, and the other accidents stated above.

The sixth Report contains cases of Femoral Hernia. In the first of these the author shews very satisfactorily that the incarcerated part of the gut is not filled with alimentary or stercoraceous matter from the parts above it, but by a secretion of its own. The consequent remarks are very judicious. We can only admit a short extract, which, we trust, will induce the reader to examine the whole,—and we can assure him he will not be disappointed.

"*Anus at the Groin.*—In my Collection there is a preparation which illustrates this subject. A middle-aged woman had a tumour in the groin, which was soft, œdematous, and inflamed. From the train of symptoms, it was obvious that this was a herniary tumour: but she would not permit the operation, nor even the approach of a surgeon. In a few days, the tumour burst, and discharged matter and feculence. She lived three weeks from the time we saw her. On dissecting the body, I saw in the labium a bag of matter, and an ulcer, with sinuses in the groin. The portion of the intestine which had been held in the sac was quite sloughed away, and the sac was no longer distinguishable. An opening, through which the little finger could be introduced, communicated with the gut, and formed an anus at the groin. On opening the abdomen, two portions of the ileon were seen tending to one point, the passage under the femoral ligament; they were in close contact, and agglutinated as they approached the passage, and adhered to the peritoneum. In the preparation, it is still observable that these two portions of the intestine have one opening towards the groin, which is owing to the wasting of the intermediate septum; and here it appears, that, if the opening had been closed outwardly by granulations or adhesions, a communication might still have remained betwixt the two portions of the intestines.

"When we look to the preparation, it appears an easy matter to pierce or to destroy that septum by either of the means I have spoken of: but let it be remembered, that, when the anus at the groin is thus established, the opening is irregular and deep. Although it may be easy to find the passage by which the fæces came out, it does not follow that the passage to the lower part of the intestine testine shall be found with the same ease. Here is an additional reason for passing the seton ligature into the extremities of the gut, in preference to passing it through the mesentery at the time of the first operation. The ligature serves to distinguish the two extremities of the gut, and, if it do not prove effectual to the formation of a communication betwixt them, it will facilitate whatever operation may afterwards be attempted. Before this simple means be rejected, let it be remembered that there is a natural tendency of the two portions of the gut to form a communication by ulceration, which appears to me to ensure the enlargement of the hole made by the seton, and its continuance."

The subject will again occur in a future report. The remainder of the present is made up of some remarks on the prostate gland, and on amputation at the shoulder-joint. The first are best suited to the meridian of Windmill-street: the last it is unnecessary to do more than announce, as such an operation can never be undertaken but by a surgeon of courage and experience, nor without a complete acquaintance of all that has been done before.

The third number contains "Pulmonary Diseases in connexion with Local Irritation, and consequent upon Wounds and Surgical Operations—Inflammation of the Lungs from Compound Fracture, and Disease of the Bladder; Inflammation of the Lungs from Compound Fracture, and Aneurism of the Aorta; Inflammation of the Lungs from Compound Fracture, and Injury of the Spine; Disease excited in the Lungs by the irritation of old Gun-shot Fractures; Inflammation of the Lungs succeeding to Amputation." Some observations follow "on the Ligature of Arteries, with some Examples of Wounded Arteries—Of the Cutting the internal Coat, effect of a loose Ligature, Ligature of a single Thread, manner of taking away a large Ligature, of cutting the Ligature short; Wound of the Humeral Artery; After-Treatment; Dissection; Observations on the Case; Case illustrating the State of a Limb when the Main Artery is torn by Gun-shot; Bleeding from returning Vessels; Wound of the Inguinal Artery, the External Iliac Artery tied, fatal from Hæmorrhage by returning Blood; Remarks on the Cases." On these subjects, we trust Mr. C. Bell will avail himself of the hints we ventured to offer in the Retrospect at the commencement of our present volume.

We have next a "Report of Cases of Wounds in which the Question of Amputation is brought forward." This is introduced by the following paragraphs:

"The wards containing, at present, cases which must have excited the sympathy of the pupils, and taught them to reflect, with great interest, upon the question of amputation, I think this the best

 time time to review the subject, in reference to the practice of the whole season. The pupils have seen, with their eyes, those things of which no words can convey a distinct impression, and which are, notwithstanding, necessary to the comprehension of this question, and to the formation of a right judgment.

"This is a subject of great extent, and embraces a great variety of diseases; but I shall throw out of the discussion the cases of wounded arteries, white swellings, carious bones, tumours, &c. and confine myself to the questions of amputation in the cases of wounds and fractures. Through the whole range of these cases, the influence of the constitution on the wound is the circumstance the most to be attended to; for, without reflecting on this—without determining how much of the character of a wound is a direct consequence of the injury, and how much is to be ascribed to the reflected influence of the constitution, a very difficult question is made still more obscure. I shall, therefore, present, in the first instance, examples of slighter injuries, aggravated by the vice of constitution, and giving rise to the question of amputation. The question will then be stated in reference to the violence sustained, as by machinery; then will follow the examples of compound fractures; and, lastly, a comparison will be instituted betwixt the compound fracture and gun-shot fracture."

On all these subjects, we meet with several useful remarks; but we cannot help thinking that in this, as in most other disputes, the best-informed men are more nearly of the same opinion than they seem to be. The author shews several points in which the army and navy surgeons would differ much less if they adopted the same language. Another thing, we conceive, is not often enough attended to:—In injuries where the loss of blood is inconsiderable, and the subsequent operation, in that respect, equally successful, we have thought that the abstraction of blood from a vein would very much lessen the danger of that rapid inflammation, which, in young men, full of animation, and preternaturally excited, so often leads immediately to mortification. It is, however, scarcely possible that this question can remain any longer in doubt, after the carnage of the late war, and the vast number of operations by well-informed surgeons from every part of the world, conducted in every climate and in every possible state of health. The number concludes with a report of the use of the nitro-muriatic acid bath in certain obscure cases of syphilis.

"When a poor creature is reduced to great weakness, despairing from long suffering and disappointment, covered with scabs and ulcers, and loathsome, and rejected of his friends;—When such an object, half poisoned with mercury, and still suffering from syphilis, or its sequelæ, presents himself, what a relief is it to be able to take a middle course; to possess a remedy which, without further weakening weakening the powers of life, can clear the skin, and dry up the ulcers, give animation and colour to his countenance, and thus enable us to return him to society. What, although witnessing such effects of a remedy, we were to be left disputing about the action of the medicine, or the nature of the disease—of how little real consequence is this difference of opinion?"

"It is not my purpose to enter upon the investigation of the fictitious diseases, as they have been called, nor to object to the names which have been given them, nor to deny the existence of new diseases; but I must express my belief, that the subject of pseudo-syphilis has acquired an importance, from the multitude of cases of syphilis improperly treated, which offer themselves in public institutions. We find practitioners screening themselves under the authority of great names, and believing themselves to be blameless, because dealing with some new form of disease, when, in fact, they have mismanaged a common case of syphilis. Whatever advantage hereafter may accrue from the new opinions, they have, in the meantime, encouraged great negligence and irregularity in the treatment.

"On slight suspicion of infection, small doses of mercury are given, which control and change the signs of the disease without curing it, and hold its virulence suspended, or weaken the attack. The improper treatment of the primary sore is another source of error. They attempt to destroy it with caustic, or they apply escharotic and stimulating dressings, while they load the system with mercury. By local applications, the hardness of the ulcer is kept up, and the mercurial course is pushed with the design to destroy the hardness;—then comes mercurial sore-throat, and they have entered the labyrinth! Instead of waiting to observe the character of a sore, and avoiding every thing which can change its aspect, they engage in a mercurial course before they have ascertained the disease. It may happen that mercury aggravates the disease, and they attribute to the progress of syphilitic poison that which is the consequence of the remedy. Another source of error is from pushing the mercurial course, when either the disease is not in a condition to be cured, or the health is so reduced as not to be able to withstand the remedy in that degree necessary to overcome the disease. The opinion that mercury will certainly overcome the true syphilitic disease, if given in sufficient quantity, leads, in the first place, to very severe trials of the remedy, and to the conclusion, that it cannot be the disease which remains or returns, after an interval of health, in a new form. The symptoms are, therefore, trifled with, and treated with small doses of mercury, which tend to suspend, and not to eradicate, the disease. Scrofulous complaints will sometimes be excited, which give rise to mistakes. Often a scrofulous swelling of the glands of the groin will be excited by venereal virus, which mercury is unable to subdue. Scrofulous ulcers of the amygdalæ occur during a course of mercury, being excited by the remedy; and these also lead the surgeon into the most serious mistakes."

A few

A few remarks follow on the use of mercury in the true syphilis, after which Mr. B. continues,

"These are some of the causes why syphilis shews itself weakened, almost worn out, but not extinguished, liable to break out in circumstances favourable to its development, but still in a state to be subdued by the lesser remedies; and, in the meantime, the patient drags a wretched existence. These, too, are some of the causes why so many patients are seen, whose constitutions have been destroyed by repeated long courses of mercury, which still seem to have been given ineffectually, since the patients are covered with sores and cutaneous eruptions."

Though we have endeavoured, as much as possible, to shorten the above extract, yet we expect the reader to accuse us of its length; and it must be admitted that too much is said unless the question were regularly argued. Pseudo-syphilis and scrofula are words which mean every thing and nothing. Eradication of a disease is a dangerous figure, and the return of the symptoms in a new form is not a return of *the* symptoms, but of new symptoms, not only in form but in place also. We admit all this would require an essay of itself, and that Mr. C. B. professes only to tell us the effect of a new remedy in certain obscure cases. To this we answer, that the title of the paper should have been On the effects of these baths in certain obstinate ulcers and ill-ascertained cutaneous diseases; and the paper itself should have been confined to informing us that we have a safe empirical remedy for some local disease which we are often at a loss how to treat. Let not the reader be offended at the term empirical. We mean it only in its proper sense, a remedy which we are justifiable in trying, without exactly ascertaining our disease or the *modus operandi* in cure. Had such been the language, as it is the true meaning, of the author, we should most unreservedly have applauded the manner in which he accuses "practitioners of screening themselves under great names," when, in fact, neither they nor their teachers have any rational, or, if they please, any object truly physical in view; that is, any inquiry into the laws of a disease, or the operation of the remedy.

A paper is added, by Dr. Scott, the author of this remedy, in which it is shewn that these baths have been useful in many hepatic diseases. We cannot question the credit of such authors, and are ready to offer our acknowledgment for the discovery of a new remedy, and its practical application. But why this unnecessary guess,—that "not a particle of the acid enters the system, and that the whole effects arise from chlorine?" Surely such a suggestion is premature. Is there any reason why these substances should not be absorbed when when the skin is broken? or what do we know of the medical effects of chlorine, the existence of which has only lately been ascertained? If the acids are not absorbed, may we not suspect that the whole benefit is derived from the fumes entering by the lungs. Let us recollect, too, what the celebrated Abbé Elesée performed with Bareges water, and afterwards with his artificial Bareges water, which was brought in "common wine bottles from the apothecaries and chemists." Here the acid used was the sulphureous. But it is time to dismiss the subject, which, however, we cannot do without wishing the gentlemen to consider the question as hitherto empirical, and to direct their experiments in such a manner as to reduce the result to certain laws, after which the practice may be dignified with the title of physical.

The fourth part commences with a most interesting inquiry—the fungus hæmatodes of that meritorious writer Mr. Hey. We might express our astonishment that so dreadful a form of cancer should so long have remained undescribed or undistinguished, were it not that only a few years before, angina pectoris was confounded with the more common forms of asthma; and that Mr. Hunter first suggested certain characters by which syphilis may always be distinguished, and certain laws by which the disease and its remedy are governed.

The cases related by Mr. C. B. are only interesting as all other tragical histories prove. They are also well told, and convey a sufficiently accurate idea of the phenomena and progress of the disease. But we cannot pass over certain objections which struck us most forcibly in the introductory remarks. After informing us, we doubt not with great justice, that the disease was well known, but inaccurately described, as well as ascribed to an inefficient cause, before Mr. Hey wrote, Mr. C. Bell cannot refrain from amending, if not the description, at least the language, "of one, than whom," he remarks at the same time, "no one can boast a more useful life."

"The term spongoid inflammation has no other recommendation than the merit of him who used it; and the name of *fungus hæmatodes* neither accurately corresponds with the character of the disease, nor serves to convey an idea of it sufficiently alarming. The bleeding from these tumours is in a great measure an accidental circumstance; and it is not the growth of a fungous tumour which is alarming, but the propagation of a disorder fatal to life.

"In all these cases which I have published, the patients were of an age and of a constitution which would have inclined me to say they were scrofulous; and this is at the same time saying that they were not of the age or constitution which we find subject to cancer.

Indeed,

Indeed, the disease has few points of resemblance to true cancer, unless it be the manner in which the tumour spreads, converting every structure into its own nature, and the manner of its becoming ultimately a constitutional disease, and attaching itself irregularly to remote parts of the body."

The objection to spongoid inflammation may be very just, and we hope it is, for to us it is utterly unintelligible; but we can see no objection to fungus hæmatodes; and, before a writer undertakes to correct the language of the discoverer of any physical phenomenon, it would at least become him to explain his own terms: what then does he mean by scrofula, and what by cancer? As far as we can judge, scrofula is a disease of a certain age, and cancer also. But the cases which follow were in subjects from 68 to 28, with the intermediate periods. This is surely a great latitude for the admission of scrofula, and would be a most desirable restriction for cancer. But "the only resemblance to cancer is the manner in which the tumour spreads, converting every structure into its own nature, and becoming ultimately a constitutional disease." If cancer and fungus hæmatodes have these properties in common, surely we are authorised to include them under a general term, marking those peculiarities in which they differ. Such is the original distinction of the word by Celsus—"Non solum id corrumpit quod occupavit sed etiam serpit." Such is the general description. Afterwards the same writer continues—"deinde aliis aliisque signis discernitur." We are not offering Celsus as an invariable authority; but it is convenient in surgery to have some one to whom we can refer, and we do not see that this language materially differs from Mr. C. Bell's.

"The meshes (we are told) of cellular texture are of all sizes. They are in smaller circles in the centre, and longer towards the circumference of the tumour. They are not like the common cellular texture, and are unlike the texture of true scirrhus and carcinoma. They have a particular density and transparency, and are in this respect like soft cartilage.

"The greatest portion of the tumour in the examples which have sent forth an exuberant fungus, consists of a mass of concreted blood."

May not such a disease be arranged under the *genus* cancer, with the *specific* name of fungus hæmatodes? Would that nosology in general could be built on so fair a structure!

We shall not dwell on the cases otherwise than to remark that they are all well related, and conclude with an accurate description of the appearances after death; and thus fill up a dreary catalogue to remind the most boastful among us of the imperfection of our art.

The

The "Report of Tumours which take their rise from the Gums and Alveoli" is judiciously drawn up, and full of information. It is but doing justice to the author to make an extract from this section.

"The nature of this tumour of the gums is not very obvious. Certainly, the worst diseases do not come from the irritation of a bad or spoiled tooth. Thus we see a carious tooth attended with ulcer and gum-boil, and abscess in the jaw; with fungous tumour of the gums, even with necrosis of the jaw. We find the inflammation from the same source amounting in severity of pain to that of the tic doloureux. But these are of no account, compared in danger with this tumour of which I am treating. This more formidable disease begins where the adjoining teeth are apparently sound, and when we cannot trace it to any common source of irritation.

"This tumour first shows itself in a small hard prominence of the gum, shooting out betwixt two of the teeth; and the teeth being good is an unfavourable circumstance, for, when they have become loose, and are displaced, without being themselves diseased, it implies that the cause is deep, and not to be removed by pulling the teeth. If the teeth be carious and originally in fault, we have a reasonable expectation of arresting the progress of disease, by removing the teeth; but when, independent of the teeth, the tumour has its origin in the membrane of the fang, or in the socket, we cannot hope to extirpate the disease, without removing the whole system of parts, the whole of what is connected in constitution. But further, the following cases prove how such tumours propagate their structure to the jaw itself; to the bones of the face; to the membranes of the nose; there being, indeed, no limit to their progress, but death. There is thus imposed upon us the necessity of performing the operation decisively, as soon as we have ascertained the tumour to be of the formidable nature here described.

"*A Tumour of the Face, originally springing from the Gum, attempted to be extirpated.*

"A farmer living in the parish of Pinner applied to me for advice, and in the expectation that I would remove a large tumour which disfigured his face in an extraordinary degree. This tumour filled his mouth, and occupied the right cheek. He told me that all the teeth of that side of the upper jaw had been successively drawn, in the hope of removing the cause of the disease. In doing this, the teeth were found loosened from the bone, and very easily detached from the substance of the tumour. The tumour had been a long time of assuming the condition in which it now appeared; but its progress had not been delayed by any thing that had been done.

"I found the body of the tumour firm, and that only the projecting knobs or lobes had a spongy elastic feel. Its lower surface in the mouth was ulcerated, and the teeth of the lower jaw were deeply imprinted on it. It had expanded in the plates of the alveolar processes, and heaved up the superior maxillary bone, so as to give a remarkable obliquity to the face.—After fully deliberating on the case, and having made sections of the bones of the face to practise what was fit to be done, and to adapt my instruments, I proceeded to the operation.

"An assistant drew back the cheek. I made a cut behind the tumour down to the alveoli. Into the slit I inserted a small saw, and cut across the alveolar process, and entered the saw deep into the body of the maxillary bone. I made a second incision anterior to the tumour and across the gums, and again divided the alveolar process. As yet there was little bleeding, for I had not cut more with the knife than was necessary for the operation of the saw. I now drew the knife along the base of the tumour on the inside, separating it from the roof of the mouth. I did the same on the other side, separating the tumour from the cheek, and cutting to the bone. Next carrying a thin and fine saw in these incisions, and moving it horizontally, I cut the shell of the bone. Finding that I could not yet detach the tumour, I had recourse to a very clumsy instrument which I had prepared, not much unlike a blacksmith's forceps, the blades of which embraced the tumour without touching it, while the sharp edges went into the jaw, already in part divided. I crushed through the jaw, and brought away the whole mass. The tumour, and the alveolar processes of that side embraced by it, and part of the jaw bone, were brought away; the antrum was opened; and there remained a great chasm: a portion of sponge, dipt in turpentine, was thrust into the space, and then a compress of lint. The lower jaw was brought up, and bound with a double-headed roller, so as to press the sponge and keep it steady. The loss of blood before this apparatus was applied had been very considerable; but the bleeding was by this means effectually suppressed. The patient, though he could not speak, gave signs of great contentment, and was put to bed with a large dose of laudanum.

"If this be thought a severe operation, I must affirm that it is not equal to what I should now think myself authorised to perform in a similar case.—The second day I took away the dressings, or rather the stuffings, and then commenced a severe process of dressing; for every day the chasm was burned with muriatic acid, and rudely brushed with tincture of bark and myrrh, and camphorated spirit. For some months this process seemed to be attended with success, and the chasm filled up with a firm cicatrix; but in eighteen months the tumour had assumed all its terrors. The side of the face was heaved up; the head disfigured; a continued dull pain attended its increase; it became ulcerated and foul towards the mouth. The patient lay much in bed, torpid at last, in part from exhaustion and irritation, and in part probably from the tumour affecting the base of the skull. He was at length carried off by colliquative diarrhœa.

"With such an example before me, you will not be surprised to see me anxious to take away the whole tumour, and the part also from which it grows."

Some

Some other cases follow, in which the author, having seen his patient earlier, was successful in his operation. This is an important chapter, nor do we recollect to have seen the disease so well described in print. Mr. Hunter, we believe, in his lectures, mentioned it under the name of the "fungating sore," and advised the actual cautery to be applied on the bone after the tumour is as much removed as the scalpel and scraper can effect.

We have dwelt so long on this performance, that we can only announce the remaining papers, reserving a few remarks for the conclusion. The papers are, "Report on Gun-shot Wounds at the Knee; Case of Baron Driesen—Report on Sacs formed in the Urinary Bladder; on Incysted Calculus; on Sounding for the Stone; and on the Method of performing Lithotomy when the Stone is sacculated—Report of Fracture of the Skull, and of the Counterfissure: being Observations on the Form and Joinings of the Bones of the Cranium, introductory to the Cases of Injuries of the Head." This chapter contains, "Experiments on the dead Body; Extravasation under the Skull; Cases of Counterfissure; Illustrations of this Phenomenon; of the Form of the Skull and of the individual Bones, illustrative of the Force to which they are exposed, and proving Design in what has been attributed to Accident." We would wish to ask who ever attributed "this formation to accident?" It is very much the custom of another class of writers to suspect what, by the help of a pun, they call the *religio medici.* But Mr. C. Bell should know better. As far at least as our knowledge extends, who have lived longer than Mr. C. B., no physiologist of this country has ever questioned design in the formation of the skull, or of any other part of the human or animal frame in general. They have not, it is true, always had the courage to ascertain what the design might be; but, where so many evident marks occurred, they have been ready to admit the prevalence of the same in all others. Hitherto we are ignorant of the uses of the red particles in the blood; but we never heard a suggestion that they were not of use. If any thing can lessen our admiration of a First Cause, it must be the obstinacy with which some deny imperfection in a fabric, the consequence of whose imperfections we are every moment called upon to remedy. There is, indeed, another mode of undervaluing the inimitable provisions of nature, which the zealots, from the best intentions, are too apt to fall into. Thus, one writer tells us of the structure of a watch, as a proof that there must have been an Artificer for the living productions of nature; and Mr.

 C. Bell

C. Bell talks to us of carpentry, groinings, abutments, and arches; tenons, mortises, and other terms, taken from the mechanical powers and their application to operations. In all this he appears to us to lessen the dignity of the object he means to exalt. Where are the arches, where the centre-pieces or key-stone, where the abutments, where the piers, of the skull? In other words, are not all these effects brought about by means which show a power infinitely beyond the application of the laws of mechanics, though those powers, in a few instances of the laws by which common matter is governed, are made to co-operate in the general design. When we hear of watch-makers, carpenters, and masons,—of their labours brought as an illustration of what they never can imitate, we are always fearful lest the allusion should be still more familiar, and the tendons of the fingers should be illustrated by the machinery in vogue with the four-in-hand gentlemen. In this last instance, the allusion is much closer; nor can we be surprised if, in their structure, a disposition of bones and tendons something like the laws of common mechanism should occur. But is this to be brought as a proof of design, when they evince only what, to a certain degree, can be imitated by man, who can only avail himself of materials unconnected with life?

It is not our intention, by this, to doubt for a moment the goodness of Mr. C. Bell's meaning; but to remind him and others of the uselessness, not to say impropriety, of using such means to prove what no man can doubt whose opinion is worth any regard.

Such are the general outlines of these Observations. That the compilation is useful, cannot be questioned; and it is with pleasure we find the author intends to continue it. We are not less pleased at his not wishing to "promise the same regularity or frequency of publication in future." We shall be ready to wait with patience, trusting that the delay will be "counterbalanced by the Reports being richer in cases and pathological inquiries, and more carefully composed." We recommend a revision, also, of some of the papers; and, above all, a more accommodating style. There is no reason or propriety in continually courting novelty of expression, or seizing opportunities of inducing the reader to believe that all knowledge is confined to one particular source. Mr. C. Bell has merit enough, without assuming more than he possesses, or without undervaluing the acquirements of others.

*Edinburgh*

*Edinburgh Medical and Surgical Journal*, No. LII.
October, 1817.

*(Continued from p. 411.)*

Art. III.—*On the Mercurial Treatment of Yellow Fever.* By J. B. Sheppard, Member of the Royal College of Surgeons in London, and Surgeon in the Royal Navy.

This paper contains a very ingenious elucidation of Dr. R. Jackson's remarks, published twenty years ago,† concerning the fevers of St. Domingo, namely, that the supposed benefit of mercury was altogether fallacious. That the common remark concerning the certainty of the remedy when the mouth could be made sore, amounted to no more than to shew that in some subjects the disease was so mild as to leave the constitution still susceptible to the impression excited by mercury, but that in the worst cases there was a total insensibility to the stimulus it usually produces. Thus, as the sagacious Sydenham remarked of the small-pox, in the severe form it is often beyond the reach of the physician; and in the mild form it is not always in the power of the nurse to kill the patient. Perhaps, in the present instance, we might say, of the doctor; for we have no doubt many subjects have been destroyed by the time lost in the use of mercury instead of the lancet, and that many, under slighter cases, have been unnecessarily tormented with a mercurial ptyalism. As a preventive, we believe some practitioners are not so much of opinion that "whatever cures necessarily prevents," but have thought that a previous salivation might lessen that high health which renders the access of yellow fever particularly formidable to new comers.

Notwithstanding the few remarks we have made, we consider this paper ingenious, well written, and replete with useful and well-digested matter.

Art. IV.—*Observations on Inflammation and Brain Fever.* By James Wood, M.D. Newcastle.

In this paper, cold affusions and bleeding are strongly recommended in brain-fever, and in the disease lately called *delirium tremens*. We have every reason to confirm the author's sentiments, and again to remind our readers of the practical remarks of our valued correspondents.

† See Outlines of Fever, Edin. 1798.

Art. V.—*Case of Stricture of the Rectum successfully treated.* By G. F. EDWARDS, Member of the Royal College of Surgeons, &c. and Surgeon at Bath.

This paper, though it contains nothing very new, does credit to Mr. Edwards's attention. Many cases of obstinate costiveness arise from stricture of or near the rectum, and, by the inattention of the practitioner, who is satisfied with the temporary relief by a cathartic as long as it be procured, such cases prove fatal, without a knowledge of the cause till all remedies are useless. Nothing can excuse such inattention. After every examination and inquiry, our art is sometimes imperfect, a consideration which should induce us to redouble our diligence, and to demand examination as often as we conceive any doubts may be removed by it.

Art. VI.—*Case of Recovery after the Separation and Discharge by Stool of a portion of the Ileum.* By ALEXANDER RENTON, Surgeon, Penicuik.

This is a very interesting case, but it will be unnecessary for us to do more than refer our readers to a similar one, related by Dr. Hull of Manchester, in our 7th vol. page 22, and to Dr. Baillie's most candid and ingenious remarks, page 104 of the same volume.

Art. VII.—*Case of Tetanus.* By EMANUEL LAZZARETTO, M.D. F.R.S. and Member of the Royal College of Surgeons in London.

This case presents nothing very new. The patient recovered; but the disease does not appear ever to have assumed its most unfavourable aspect.

Art. VIII.—*Case of obstinate Colica Stercorea, cured by the exhibition of Quicksilver.* By JOHN O'NEILL, Surgeon, Fermoy.

This history, as the judicious author observes, shows that in cases of obstinate costiveness, with pain, and succeeded by stercoreous vomiting, the empirical use of crude quicksilver is not only justifiable, but becomes a duty. We should have been better satisfied if, in the beginning of the disease, or as soon as the patient (who is described of a full habit and sanguine temperament) applied for relief, venæsection had been among the remedies, if not the first.

Art. IX.—*Historical Sketch of Medicine in the Russian Empire, from the earliest period to the present time.* Communicated by Dr. VON EMBDEN, of Hamburgh.

Art.

Art. X.—*On the Benefit of Cold applied to the Head in the Fever called Typhus.* By J. Wood, M.D.

Art. XI.—*Case of Femoral Aneurism cured by tying the external Iliac Artery.* By Archibald Robertson, M.D.

On a careful perusal of this case, we could almost say we were convinced that the artery was obliterated by pressure, and that the subsequent operation was altogether unnecessary. The whole is, however, so intimately connected with a subject to which we have lately paid much attention, that we shall reserve it as the basis of a future essay.

Art. XII. contains an affectionate tribute to the memory of our late colleague Mr. Royston, from Mr. Robinson Scott, a Surgeon of the Royal Navy, and Fellow of the Linnean Society.—Of Mr. Royston we may truly say *nobis gratissimus*. Having already given several particulars of his life, and the circumstances of his death, we shall only transcribe the close of this article, as an useful lesson to ourselves and all our brethren. After mentioning Mr. Royston's preparation for a "Bibliotheca Medicinæ Britannicæ," his printed prospectus, and the manner in which it was noticed by Dr. Young and some others, Mr. Scott adds,

"An anonymous writer in the Antijacobin Review for June 1808, among other observations on Mr. Royston's publication, quaintly elaborated the following, viz.—"We do not know any greater use of *Bibliothecas* than to foster indolence, generate vanity, and increase pedantry and superficialness. Such works are never encouraged in any country, unless before the public have attained a taste for inquiry, or after they have lost it.' And again, 'Mr. R. appears to possess talents adequate to the task in which he is engaged; and, if we can induce the people of the United Kingdom to depend more on temperance than on drugs, for the recovery or preservation of their health, we shall wish his work every possible success.' Such is the *superficial* flimsy jargon of a conceited reviewer, on which it is hardly worth while to waste any remarks. It is manufactured in the usual style of the minor critiques in the common run of inferior miscellanies, which, by a sweeping assertion, or flippant paragraph, *appear* to do great things, and perhaps leave on the idle portion of their readers a strong impression of profound erudition and oracular dignity. The '*slashing Bentley*' of the review above mentioned also says, 'On the decline of states, we usually find their philosophy and literature minced down into dictionaries and *Bibliothecas*.' Now, this last sentence is mere nonsense. In a note, in Mr. Royston's hand-writing, on the margin of this review, now before me, is this remark: 'As states rise into a high degree of civilization, their literature and science increase and accumulate; and, *in consequence*, Bibliothecas become useful and necessary.' This critic, whoever whoever he is, does not know that the character of a *Bibliotheca* comes nearer to that of a REVIEW than to a *Dictionary*. In fact, it should be a good review, on a great scale, extending, in a certain department of science, back to a period of publication long prior to the institution of critical journals.

"No one can doubt, however, notwithstanding the palpable examples exhibited in many reviews of inadequacy to their task, or of characteristics worse than this, of their *general* utility. Numbers of them certainly exhibit the most glaring instances of the abuse of a thing *in its principle good.* Medical critics should, most especially, keep clear of such blemishes. When, indeed, vast quantities of medical books issue from the press, neither containing any thing new, nor exhibiting any thing old in a new light; adding to the heavy expence of study, *ostensibly* original works, but covertly only ponderous and circuitous *advertisements;* preceding, not consequent on practice;—the *intended cause,* not the valuable result of professional experience:*—it is then that the medical censor should speak out, forcibly and firmly. To stop, or, if he cannot stop, to divert the wordy torrent, to point out *only* what is excellent to the student, to whom money often may be, and to whom time always ought to be, most valuable; he must mark, with decided disapprobation, literary quackery and pseudo-philosophic imposition, whether of *native or of foreign growth;* and discriminate between productions likely to facilitate the anxious progress of the student, and crude compilations fitted for the mere 'helluo librorum.' He should separate what are real improvements in science from scholastic hypothetical *dicta,* too often palmed on the world under the semblance of system. To do this is to do right; but the task is not easy, nor the gratitude of the public very abundant for the performance of it. It is become, however, an indispensable duty, and if, in the discharge of it, the critic acts from pure motives, positive utility to science may be the result of his efforts, and he will have for consolation, at the least, the 'mens sibi conscia recti.'

"But whilst, on the one hand, medicine has to lament the *quackery* of book-making, she is, on the other, often compelled to regret the loss of much excellent practical observation. Many practitioners of celebrity, who have obtained the 'otium cum dignitate,' consequent on long success in life, ought to be less reluctant in giving the world condensed views, at least, of their extensive experience. Few such works as those of HAMILTON and HEBERDEN appear *in an age;* and yet many eminent physicians, of practice equally extensive, too churlishly withhold from their brethren the results of their truly useful labours. Satisfied with having basked in the very sunshine of practice, it is to be regretted, that they who have, as it were, for a series of years, been absorbing so much of the light of

"* See also excellent observations on medical book-making, by Mr. Royston, Med. and Phys. Journal, vol. xxiv. p. 2."

experience, experience, should not reflect *some of its rays* upon the less enlightened of their contemporaries.

"I now conclude this paper. It may be said by many, that in some places I have used too much the language of panegyric. It may be so, perhaps it is so. For the general strictures included in these observations I offer no apology:—'Licet omnibus, licet etiam mihi, dignitatem '*Medicinæ*' tueri; potestas modo veniendi in publicum sit, DICENDI PERICULUM NON RECUSO.'"

---

*The Morbid Anatomy of the Brain in Typhus Fever, with a few Observations on its Nature and Mode of Treatment.* By THOMAS MILLS, M.D. Licentiate of the King's and Queen's College of Physicians, Dublin, 1817. Pp. 26.

THE following preface introduces twelve cases of fever, with the appearances after death.

"While an epidemic typhus, fatal, especially among the higher orders, spreads general alarm in several parts of this country, whatever throws light on the nature of that disorder cannot fail to interest the public mind.

"In a work, written expressly on the subject of Fever, I proposed venesection and evacuants as the best remedies in typhus, and gave numerous cases, from my private as well as public practice, in which these remedies were used with advantage.

"This mode of treatment has since received the sanction of practitioners of eminence; while others, without adopting it themselves, have been disposed to allow, that it is not attended with any injurious consequences.

"But, besides my own experience and that of others, I think it proper to appeal to a test, no less decisive, as to the merits of any practice—the morbid changes which appear in the bodies of the dead. An examination of these changes, as Dr. Baillie, in his Morbid Anatomy, observes, is calculated *to correct theories too hastily taken up about diseases*.

"I recommended venesection on the ground that the disease, though attended with extreme debility, proceeded from inflammatory actions in the brain. If this be *a theory taken up hastily*, or from erroneous views of the subject, dissection will detect its errors; but, will, on the other hand, give it additional strength, if it be founded on just observation and in truth.

"The following sheets present twelve cases of dissection, shewing the morbid appearances of the brain in typhus, or brain-fever."

We transcribe only one of the cases, as, with a few exceptions, chiefly in degree, they are all similar.

"Mrs. ——, aged 32,—Townshend-street,—of a melancholic temperament, and subject to a lowness of spirits. Nine days ill of fever, caused by suppression of the menses and exposure to cold and wet, wet. Shivering, pain and fulness of the head, languor, loss of appetite, and a soreness of the flesh and bones, ushered in the attack. The pulse was frequent, irregular, and variable in strength; the skin was hot; there was delirium, throbbing of the temples, coma, deafness, and hurried respiration; there were involuntary dejections: the body was covered with petechiæ, and there was a loss of speech and the power of deglutition.

"The patient died on the 15th day of the fever.

"On dissection, several distinct osseous tubercles were found along the line of the longitudinal sinus; the dura mater was highly vascular, and adhered closely to the cranium; the veins of the pia mater were turgid, and there was a considerable degree of arterial vascularity throughout this membrane, particularly at the posterior part; the arachnoid membrane was raised from the pia mater by a serous effusion, principally at the anterior portion of the brain.

"On cutting into the substance of the cerebrum, it exhibited numerous red points, but the cortical part was somewhat paler than usual. The plexus choroides were more vascular than natural; about twelve drams of a watery fluid were found in the ventricles and at the base of the brain. The cerebellum was still more vascular than the cerebrum.

"The right lobe of the liver was rather darker and harder than usual, and the left was adherent to the diaphragm; but its substance, when cut into, was of a natural appearance. The pancreas was gristly, hardened, and enlarged; the right ovarium was altered in its structure, and, when cut into, exhibited matter of a cheesy consistence; the left ovarium contained a large hydatid.

"The thoracic viscera were healthy."

With the exception of the bony tumours, such was the appearance within the cranium of each, excepting that in some the quantity of serum was greater, and in others coagulated lymph was discovered in different parts of the plexus. In some of the subjects, also, there were appearances of inflammation in other viscera. There is no account of the manner of treatment in any, and unfortunately no account of the period after death at which the examinations were made. The last is of no other importance than as a means of conjecture how far the fluid found in the ventricles was the effect of disease, or of transudation after death. Other desiderata in the account are, a proper description of the petechiæ during life, and whether the blood coagulated after death. Only one of the subjects had *delirium ferox*.

Having related his cases, Dr. Mill's concludes,

"The above are twelve cases of typhus fever;—the debility, the head-ach, the derangement of the mental powers, and the petechiæ with which the body was covered, sufficiently determine the nature of the complaint. The dissections were conducted by surgical gentlemen of character; and the descriptions of the morbid appear-

 ances, ances, taken down on the spot from their report, were confirmed by my own immediate examination.

"These appearances were almost uniformly the same;—vessels gorged with blood, extending themselves through the substance of the brain, overspreading its lining membranes, the dura and pia mater, and the arachnoid coat; and effusions between these membranes and into the cavities of the brain. These are analogous to the changes observed in phrenitis, in hydrocephalus, in apoplexy, and similar to those which are found in the cavities of the chest or abdomen, after a fatal pleurisy or peritonitis.

"While we pronounce, without hesitation, that, in these cases, the changes are indicative of increased and inflammatory actions in the organs in which they are discovered, can we suppose in typhus fever, where analogous changes take place in the brain, that the same disordered actions do not go forward?—especially, since the appearances on dissection, by shewing the relation between the symptoms and the organic changes produced by disease, are explanatory of the phenomena.

"It is true, great debility occurs; but, when the brain is oppressed and deranged by excess of action, that consequence is to be expected, since the oppression is exerted on an organ which is the very source of muscular energy: and hence, in the apoplectic, where the oppression arrives at its height, the powers of the voluntary muscles cease altogether.

"The effects of stimulating medicines in such a state of the brain must be to urge the blood with still greater violence on vessels already overgorged, and to increase action in the exhalents which are disposed to inundate the cavities with their exuding fluids.

"On the contrary, whatever lessens the strength of the circulation, the very same remedies which are administered in phrenitis, seem to be called for here—sedatives, purgatives, and venæsection, employed and repeated according to the state of the symptoms.

"Such is the conclusion which, I think, the unprejudiced would draw, and it is supported by my experience and by that of practitioners who have adopted the practice in the present prevailing epidemic.

"Among other communications, I have been favoured by Mr. Henry, surgeon, who has the care of the Dispensary at Castlepollard, with an account of the epidemic fever, as it appeared in that town and its neighbourhood.

"'The symptoms,' he says, 'were chilliness, pain in the head and back, low and quick pulse, weakness, petechiæ, delirium, stupor or heaviness in the head, foul tongue, thirst, and restlessness. Recoveries generally took place from the 9th to the 13th day.

"'Contagion appeared to be the cause, as it attacked successively several in a family. My apprentice and I blooded nine on the same day, in one family of the name of Walsh, two miles from Castlepollard, on the estate of Thomas Webb, esq.

"'From the dispensary books it will appear that there were about 200 ill of the fever, all of whom were blooded, and not one

 died.

died. Some were blooded three times: one of these was my apprentice. The quantity of blood taken at each time was from six to eight ounces from adults—less from children.

"'I had several patients not on the dispensary books, and I treated them in the same manner, and with the same success: I also employed purgatives and sudorifics.

"'The head was invariably relieved by each bleeding. I found that small and repeated bleedings were more effectual than large ones.

"'The blood was seldom buffed, but generally dense. The fever terminated sometimes by perspiration, but often without any sensible evacuation, with a return of sleep and appetite.'"

Such are the important facts contained in this valuable compilation, on which we cannot help making a few remarks.

First, we have still to learn what constitutes typhus. Is it contagion? The author seems rather to insist on debility and the other symptoms. Now, all these symptoms may occur in scarlatina, measles, or small-pox; or in continued fever from any cause. That the brain was oppressed in these, and is in most or all other, fevers, cannot be questioned. But, when we are told that the appearances are similar to those observed after phrenitis, hydrocephalus, apoplexy,—and that such changes are similar to those found in the cavities of the chest and abdomen after fatal pleurisy and peritonitis, we must pause before we admit that effects from such causes are similar. In phrenitis, we have often preternatural strength during life. In violent inflammation of the brain, we have epileptic symptoms, attended also with preternatural muscular strength for a time. In apoplexy, we have entire or partial loss of sense and motion. In the first, we find, after death, strong adhesions of the membranes, with effusions of coagulated lymph. In the second, a peculiar firmness of the brain, probably the effect of effused coagulum. In the third we have extravasation of blood in the ventricles between the convolutions, or, more commonly, under the dura mater. In phrenitis, the blood drawn is usually buffy and cupped. We take no notice of the turgid appearance of the vessels of the brain, as nothing can be more uncertain.

Now, it is to be remarked, that, where the blood was drawn in this fever, the appearance, so far from denoting inflammation, was directly the reverse: "dense," which, we conceive, can only mean of a creamy consistence, the constant mark of blood which coagulates imperfectly, as if from the same diminished power which it partakes with the solids. At the same time, it cannot be questioned that there was effusion on the brain. This, we conceive, takes place in all fevers, and to be the cause of that dulness of the the senses which the best writers have considered as favourable in a certain degree. Deafness is the sense generally marked, because it is in an organ which is principally wanted during disease.

But, if it is the property of every fever to induce this effusion on the brain, there cannot be a doubt that such a process should be closely watched. When it extends no further than to produce a general dulness of the senses, such an effect may be desirable; but, wherever a new action is set up, it may be carried further than is consistent with health. Hence, this effusion may extend to extravasation, in which case we have apoplexy, by no means uncommon in the very commencement of some of the worst kind of camp-fevers. It may induce inflammation, in which case we have *delirium ferox;* or, without either, it may be so considerable as to produce a greater interruption to the necessary functions of the brain than is consistent with the maintaining of those actions by which life is supported. Under any of these circumstances, the indication is to determine less blood to the head, which must be accomplished either by lessening the action of the vessels about the brain, by taking away blood from the common mass, by other evacuations, or by exciting higher action in other organs. The first is accomplished by cold applications to the surface of the head, the second by venæsection or cupping, the last two by purgatives; and, if the symptoms are threatening, all three may be necessary.

But, neither the symptoms in their ordinary form, nor the advantage derived from these remedies, convince us that inflammation of the brain attends every fever; or that a limpid effusion to such a degree as merely to obscure the senses is not salutary in fevers. At the same time, we perfectly agree in the practice; and are convinced that the omission of early evacuations, and, still more, the use of stimulating remedies have produced all the mischief of which they are accused, and of which the most zealous Cullenians are gradually convinced.

---

*A View of the Relations of the Nervous System, in Health and in Disease; containing Selections from the Dissertation to which was adjudged the Jacksonian Prize for the Year* 1813. *With additional Illustrations and Remarks.* By Daniel Pring, Member of the Royal College of Surgeons, London, and Surgeon at Bath. 8vo. pp. 256. Callow.

Irksome as disquisitions on the brain and nerves usually are, we could not withhold our attention from a paper which received the prize from such an ordeal, and had been revised for for four years after by the author. We acknowledge that we found our task often irksome, which may in part be imputed to the great pains taken by the writer to compress what was familiar to himself, though new to some of us. Whilst, therefore, we endeavour to give the spirit of the work, our readers must be aware how difficult it is to compress what is intended only as a selection.

After a short chapter on the structure of nerves, another succeeds on their power of contraction when divided. This the author proves, by a very satisfactory experiment, is not the effect of mere elasticity, but of the vital power. He observed also, by similar experiments, that the same power exists in the spinal marrow.

The next series of experiments satisfactorily proves, that a punctured or divided nerve may be restored to all its functions, and probably to its original texture. Whether this is by an approximation of the divided parts, or by an union of them from a newly-formed part, does not seem ascertained. The latter, however, seems the most probable result of the author's observations; and we think, we somewhere recollect, that, when Mr. Hunter not only divided, but dissected out, part of a nerve, he afterwards found the lost substance restored, first by the union with coagulated lymph, which was gradually converted into the same substance of the nerve.

Mr. Pring next considers *the Relation of Nerves with their Centres*. By this term he designates the structure which forms a medium for the union of nerves of different distributions. Some have a relation to each other by the medium of the brain; others of the spinal marrow. Hence the sympathies of various parts which are destroyed, if their centres, or the nerves by which each part is connected with those centres, are destroyed. Thus an injury to the leg induces an action in the arm, and *vice versâ*; but, if the sciatic nerve, the spinal marrow above the lumbar vertebræ, or the axillary plexus, be divided, that reciprocal influence which naturally exists between the muscles of the leg and of the arms would be destroyed. This leads the author into some inquiries concerning the seat of sensation from the wound of a part, whether in the brain or in the part itself, and also into the doctrine of vibrations. The first, we conceive, never can be accurately ascertained, or else that it is a mere dispute about words. The doctrine of vibrations is refuted by the result of every experiment of the author's, which proves that there is no motion in the nerves, not even elasticity, without life. The doctrine, indeed, has gradually fallen into oblivion, since Haller and Mr. Hunter have taught us to to consider life and its actions as the only means of accounting for whatever we see in the physiology of animals.

A chapter on the Relation between Nerves shows, that where the nerves are double in the same organ, the division of one set induces a greater power in the corresponding set, provided the divided nerves are prevented from uniting. The experiments in proof are judiciously selected, well conducted, and the inference fair.

"I exposed (says Mr. Pring) the lower portion of a sciatic nerve (five days after it had been divided, and some recovery of its powers was indicated) for the extent of an inch: it was cut, pinched, and lacerated with the forceps: the animal did not evince the slightest sensation, but he started if the superior portion was in the least degree irritated.

"June 5. I applied two ligatures on the sciatic nerve of a rabbit, and divided it between them. I preferred the ligature to a simple division, in order to provide against a speedy re-union, which would have frustrated the design of the experiment.

"August 5. A considerable improvement of the voluntary powers of the limb had taken place; so great indeed, that I was fearful my care to prevent a premature re-union had been without success; but, in this suspicion, I was mistaken. I exposed the parts of the same nerve, and continued the dissection near two inches below the place of its division. I then applied a ligature to the most inferior point of the lower portion, but the animal did not evince the slightest evidence of sensation: the nerve was divided by the scissars with the same result. If the superior portion was pinched, or irritated, a lively sensation was manifested.

"This experiment appears to be as conclusive as any which will readily suggest itself, on a very important point of the pathology of the nerves: without such a decision, every thing which we can devise for the improvement of the practice which has been instituted for the cure of some diseases of the nerves, must be futile and nugatory; for if it should be proved, that the sensibility of a nerve is acquired below the place at which it is intercepted, by communication with sound nerves, we have but little to hope from the success of a project, which is calculated to prevent permanently the transmission of central influence. But if the improvement of the faculties of the nerves had taken place by derivation of nervous power from sound nerves (in some measure analogous to the effects of the anastamoses of the arteries), the sensibility would surely have been excited by the application of a ligature, which never fails to produce exquisite pain in a nerve which is not deprived of its sensibility.

"We must conclude, therefore, (if the sufficiency of these data be admitted) that the improvement of the nervous function of a limb, previous to the reunion of one of its divided nerves, results from the assumption of an increased power or energy, or an increase of the properties, belonging to the sound nerves. It is sufficient for the general purposes of this branch of surgical pathology, that we can positively positively deduce this inference in regard to the sensibility possessed by the nerves: it is probable that the same conclusion may be extended to all the other faculties belonging to these organs, which are derived from their respective centres."

Some remarks follow on the relation of nerves with muscles. These, in our present improved physiology, need only be mentioned. In England we grow tired of inquiring into the nature of muscular motion, and are satisfied with tracing the facts in order by them to learn the laws without attempting to dive into the causes; and the inquiry would probably cease, were it not for the institution of certain prizes from endowments made in less enlightened days. The continentalists are slowly learning to do the same; but they cannot divest themselves of some mysticism in language. Highly as we esteem Bichât, we never read him without feeling a wish that he had never imbibed the mechanical reasoning of his countrymen or that he had checked the sallies of his imagination by the physiological simplicity of Mr. Hunter.

"According to Bichât, (says Mr. Pring) a voluntary muscle displays four kinds of contractility: animal contractility, as when it acts at the instigation of the will; sensible, organic contractility, as by the action of a chemical, or other extraneous stimulus; insensible, organic contractility, as by the impulse of its fluids, constituting tone; contractility of tissue, as by a transverse section of its fibres, arising, as it is said, from failure of extension."

If this be put into common language, it can mean no more than that a muscle contracts by the will, which is voluntary motion; by its living powers, and without the concurrence of the will from local stimuli, as when a part is pricked or otherwise stimulated. The two other kinds of contraction are not so readily comprehended, and, perhaps, on that account may be more agreeable to a certain class of readers. The first, however, means no more than that plumpness, as it is usually called, which distinguishes youth and health, and is more particularly attended to in the cheeks of both sexes, and in the scrotum of male infants; the last scarcely deserves notice. Indeed, the whole paragraph is given by the author and transcribed by us with a view of showing the state of science on this particular subject. Mr. Pring next offers a very few remarks on the relation of nerves with the lungs. These go only to show, that all voluntary action of the muscles, by which the lungs are inflated and emptied, cease as soon as their communication with the brain is cut off. The subject of artificial breathing, and the generation of heat will be considered hereafter.

The relation of nerves with the heart contains many excellent observations. After a fair analysis of all that has been said said of the dependance of the action of the heart on the nerves, and of its continuing to beat after decapitation, the author with much prudence concludes—

"As every physiological inquiry has for its most important end the development of the relations which obtain in the economy of the human subject, so it is necessary, in prosecuting the investigation, to confine the researches to animals which bear with the human subject a perfect analogy in the circumstances which form the topics of the inquiry. We cannot extend the observations made upon cold-blooded animals to the phenomena of the mammalia, because the diversity of the laws to which they are obedient, is the proof of the absence of that analogy upon which alone the argument can be founded. Shall we conclude that the nervous system is not necessary to sensation, or the muscular structure essential to motion, because the zoophytes, according to Cuvier, display the faculties both of sense and motion, although they possess neither fibres nor nerves?*

"We perceive by this analysis, that much has been attempted for the understanding of the mode of the action of the heart; that some of the particulars which have been investigated, are still undetermined; and that many more remain, which have not yet been submitted to the test of experimental inquiry."

The truth is, all we can infer from the experiments of Bichât and others is, that after decapitation it is some time before the parts die, though the actions by which life is supported are so prevented as never to be restored. As long, however, as the divided parts retain a living principle, so long will they obey their accustomed stimuli. The stimulus from the will is superseded for ever; but the action of the lungs is the stimulus to the action of the heart. As long, therefore, as the lungs are made to act, or are alternately filled and emptied, so long will the heart continue its action, provided its life continue. But, after the body has suffered such violence, life must be gradually extinguished. Hence, we find the secretions cease, and the generation of heat with it, and, by degrees, life ceases, sooner or later, according to a variety of circumstances attending the animal, and the injury he has received. After this, no stimuli can excite any action. It is the want of attention to these circumstances, principally the mode of dying, that bewildered so many experimenters.

This doctrine is still better illustrated in the succeeding chapter on the "Relation of Nerves with Arteries." By some well-constructed experiments, the author shows satisfactorily the futility of many others, from which hasty conclusions are drawn; and convinces us, that, by habit and long

"* Anatomie Comparée, tom. i. p. 27."

reasoning on the subject, and every necessary improvement in conducting his experiments, he has acquired a superiority in his inductions over many who fancy they have devoted their lives to physiological discoveries.

"That the action of the arteries of a limb (says he) is not dependent upon the medulla spinalis, is proved by the fact, that the pulsation of the arteries in the fore-leg of an animal will continue after a division of the axillary plexus.

"It is also demonstrated, that the secretions, and the processes of regeneration, will likewise proceed below the place where the communication of nerves with their centre is intercepted. A testicle has been known to suppurate after the division of the spermatic nerves. But, as I was desirous of acquiring a more precise information on this point, than I had been able to possess by report, I divided, with as little injury as possible to the surrounding substances, the nerves of the axillary plexus of a rabbit, at a distance of about three-fourths of an inch from the ribs: I was particularly careful to divide every filament of nerve; and, as the operation was familiar to me, I believe that I succeeded. The limb lost its sensibility, and was rendered incapable of motion; the circulation was, however, maintained in it.

"The wound, which was principally below the point at which the nerves were divided, went through the same stages as I had observed in the same animals, under other circumstances; that is, the skin sloughed to a trifling extent; the wound became covered with a whitish concretion, it granulated, and healed in less than a fortnight. At this period, the leg was diminished in size and paralysed; but no remote destruction of integument had taken place, and the organic life was maintained. Finally, the animal recovered the use of this as well as of the other legs.

"It is thus proved, that the influence from a central termination of nerves is not necessary to the organic processes, which were in this experiment observed to take place. I am acquainted with no instances in which this influence is in any degree indicated to be necessary to secretion, except the experiments of Mr. Brodie, before adverted to; in which it was found, that arsenic produced no secretion from the mucous membrane of the stomach and intestines, after a division of the eighth pair of nerves. On these experiments it must, however, be remarked, that, if foreign agents are employed for elucidation of a natural connexion, the results, being liable to be modified by such agents, will not at all times be conclusive on the points to which they relate,

"But, although the brain does not appear to be in all instances necessary to secretion, yet the results of this process are variously influenced by the affections of the brain. Of this fact, we have many examples: a passion of the mind is capable of influencing the secretion of the kidneys, as well as that of the intestines; and, perhaps, the liver itself is not exempt from the same kind of agency; the appetite for food, when stimulated by the sight of the objects of its desire, will increase the secretion of saliva. If we admit no conclusion, which is not supported by the testimony of direct fact and experience, we cannot pronounce that the influence of a nervous centre is necessary to any of the processes of organic life. We have proofs that these processes are liable to be modified by certain conditions and affections of the brain; but, that the dependance is in the relation of a source with a channel of distribution, we have no absolute proofs; our testimonies amount only to this evidence, that these are the effects (viz. those which have been noted from a sensible demonstration) of a division of nerves. Such appears to be the condition of the evidence which relates to the connexion between remote organic life and the central termination of nerves.*

Though we find a great many good things in this chapter, yet we cannot help thinking the author has unnecessarily involved himself in mechanical and chemical causes, especially when he ascribes inflammation to any other cause than the actions of life. Perhaps we shall be told, that all the "chemico-hydraulic agencies" of which he speaks, are "directed by the principle of life, to which a preternatural condition is superadded." This means, if we understand him, that they arise from the actions of life changed according to the nature and properties of certain stimuli; and that all such changes, or such preternatural conditions, being different from the original or healthy actions, constitute disease. All this is extremely intelligible, and we heartily wish he had stopped here, by which he might readily have explained some difficulties, which, as they are insulated in notes, we shall transcribe. The first is as follows:—

"I am inclined to think, that there is a property of preserving for a certain time the fluidity of the blood, belonging to the vascular system, independent of motion; and that this property is one dependent upon the nerves. The first part of the position is, in a great measure, confirmed by the formation of a clot in the *sheath* of a divided artery, which would no more take place in the sheath than in the vessel itself, if the fluidity of the blood were wholly dependent upon its motion; for this motion continues while the coagulation is taking place. The coagulation of the blood is, in this instance, accounted for, by supposing that it becomes entangled, &c.; which is a very entangled sort of a supposition, because it flows in a space, and through this same space it may continue to flow, if its fluidity were maintained by its motion. This proof is not perfect, because the fact is not unquestionable; at least, the assigned order of its occurrence is not unquestionable. With regard to the second part of the position, the reasons which affect it are mentioned elsewhere."

---

"* This expression, 'the central termination of nerves,' is frequently employed; and I adopt it, after Reil, because the denomination appears to me good for the purposes of distinction."

 The

The second note.

"I have remarked it only in two cases of inflammation; one of them has been already mentioned, the other was as follows: A woman, when rubbing a deal table, forced a splinter of wood half an inch in length under the nail of the second finger; it produced intolerable pain and rapid swelling: about three-quarters of an hour after the accident, I extracted the splinter, and, upon examination, found the arteries which supplied this finger beating 104 strokes in a minute, while the radial artery was beating only 92."

The third.

"It is not the mere cessation of life which produces a slough. I believe that under some circumstances of the identity of this influence which is exerted by the nerves, a kind of inverse operation takes place upon the subjects of its former alliance, and that the slough so produced is essentially different from that putrefaction which succeeds to ordinary death; that it is, in short, a state of the sensible textures, which no other agent in the universe could produce, save that principle of life, and which the principle itself can produce only under one condition of it. After ordinary death, the integrity of the textures may be a long time preserved; a slough may be formed in a few hours after the influence of a cause whose relation is with the principle of life."

What connexion have these properties with hydraulics or chemistry? They are all of them the properties of life, which cannot be imitated by the application of any other laws. This is well enough illustrated in a passage in the text, which we shall transcribe, in order to relieve, as far as we can, this intelligent author from a difficulty we did not expect would be started in such a quarter.

"The same principle (says he) not only regulates the calibre, but the cohesion also, of vessels: in acute inflammation, that property of the principle which prevents rupture of the arteries is generally increased, as the vessels in this state bear a great degree of distention: we have, however, an analogy for this in the arteries of the penis; we may therefore presume that the natural strength of the property is not diminished in inflammation. In the local determination of blood to which apoplexy succeeds, the force of this property must be diminished; because I have seen a man, who had two attacks of apoplexy, sustain phrenitis without such effect. This distinction, which has been deduced from my own experience, is very similar to one of Mr. Hunter, who supposed that in phlegmonous inflammation there is an increase both of the action and of the power of the vessels; while in inflammation which terminates in mortification there is an increase of action, but not a corresponding increase of power:* by this word 'power' (than which no expres-

"* Treatise on the Blood, p. 8."

sion sion can be more vague), I understand the property of the organic spirit, by which the cohesion of the fibres of vessels is preserved."

We need hardly remark, that every action must be sustained by a corresponding power; and the only power we know of in the living body is the power supplied by life. That this is greater at one time than at another, every man knows by his own experience. It is not less certain that, under particular circumstances, actions are accomplished to which the whole body, or certain parts, is *unequal* at other times. On such occasions, therefore, the power must be increased. From the nature of a living organ, this increase of power can only be supported to a certain degree; and, if action still continues, it must now suddenly cease. Thus we find, that, after violent bodily exertions, the muscles suddenly cease to act; and, if that cessation extends to the heart, syncope sometimes follows, from which the subject does not always recover, or recovers very slowly. If this increased action is confined to a part, the powers of that part are increased; but, if the action continues beyond what the powers of that part can support, death must follow. But this death, being only partial, is effected by a new action, in which all the lymph in the vessels, having first disengaged itself from the serum and red particles, coagulates: the parts themselves are also immerged in the same substance, which instantly coagulates. Thus the parts not only die, but, in the last act of life, probably more powerful than any that preceded it, form themselves into a slough. By this mode of death, the vessels are secured from hæmorrhage, which would otherwise follow the separation of the dead part. Now, all these actions, even to the mode of dying and the separation of the dead part, are the effect of the living power, and cannot be imitated, though they may be induced by artificial means. That the nerves have a share in all these actions, we cannot doubt; but then we suspect that they have no other share than as *chordæ internunciæ*. If this continuity of sensation is prevented, all sympathy ceases; and many of the actions which are necessary for the nourishment and restoration of parts cease with it.

A section on the Diseases of Nerves follows. The first of these, as we might suppose, is the *Tic doloureux*. This chapter, which occupies 40 pages, is extremely well constructed. The experiments are well designed; the result of each related with equal minuteness and candour; and the inference from the whole such as impresses us with a high sense of the author's accuracy and fidelity, and with a proportionate estimation of his labours. If, as we suspect, Mr. Hunter somewhere remarks, that divided nerves unite not by a growth from from the divided ends, but by a conversion of coagulated lymph into nerve, we shall not be surprised if Mr. Pring found all his endeavours at preventing a re-union, and consequent return of the disease, ineffectual. We will venture to suggest one other, which can only be accomplished where the nerve is not very deeply seated. We mean to remove the whole of the substance to below the part of the nerve taken away. Perhaps it may be as well to preserve skin enough to procure the healing by the first intent, or at least that may be tried, and, if not effectual, the incised wound may be left to granulate and cicatrize in the ordinary way. A short practical paper on "Tumours of Nerves" closes this section.

The third section, "on the Effects of external Injuries of the Nerves," closes the volume, and is by far the most valuable part to every practical purpose. The previous matter is, indeed, as we might expect, chiefly introductory to these most important conclusions. The first chapter, on Inflammation of Nerves, contains more well-directed experiments, fair inductions from them and from the histories selected from other quarters, than we have met with in any other work. Not that the experiments are unnecessarily multiplied; but, from the clear conception of the author, they are all directed to the object in view, and the inference from each is undeniable. If the general result furnishes less information than we flattered ourselves with receiving, we are bound to impute this to that judgment and sense of rectitude which is perceptible in every part of the work. We every-where find experiments constructed, not to confirm a doctrine, though, doubtless, that must often have been the author's wish, but to inquire into the validity of his own or of other people's conclusions.

The last article is on Injuries of Nerves producing Spasms. In this the author has tetanus, or trismus, principally in view. The subject commences with a long quotation from a paper of Sir Everard Home's, which is quoted more at length than was necessary, principally lest any suspicion of misrepresentation should be attached to the relation. The case is certainly interesting, and does credit to the writer. The injury first received was by pressure on the thumb. The part healed, and for two years after no inconvenience was felt, excepting that the thumb was not always under the voluntary direction of the patient. During the third year, when in a post-chaise,—

"A cold wind blew directly into the carriage, and he endeavoured to pull up the window; but, not seeing the glass rise, he looked down, and his hand, instead of pulling up the window, was lying upon upon his knee. The thumb was bent in towards the palm of the hand: a spasm came upon the muscles of the arm, making them bend the elbow, and immediately he became insensible: in a quarter of an hour he perfectly recovered himself. Some hours after, upon bending his thumb, to shew what had happened to him in the carriage, there was a return of the same attack, which also rendered him insensible for a few minutes."

No ill symptoms returned till more than two months after, when, eagerly waving his hand, a contraction seized the patient, and he suddenly fell to the ground in a state of insensibility. From this time, these events occurred frequently, without any apparently exciting cause: the spasms were, however, in a certain degree, arrested by a tight ligature; and, as they were generally preceded by universal uneasiness, a tourniquet was always ready to be tightened on the first alarm. Electricity was found useless.

From the effect of pressure, and its interruption of the continuity of action, it was presumed that a division of the nerve might produce a more powerful effect. This was accomplished where the fibre passes under the annular ligament of the wrist to the thumb. The retraction of the cut-ends of the nerve was much greater than expected, as the nerve had been previously detached from all its surrounding connexions. A temporary universal spasm succeeded this division; but, for eight hours afterwards, no spasm occurred. In fifteen hours the spasm was general, excepting that the brain was not affected. The wound not healing by the first intent, the callosity of the cicatrix became a source of uneasiness, and the spasms continued with little alteration. They were somewhat abated, as that hardness was absorbed; but returned in such a manner as to prove the inefficacy of the operation, which Sir Everard imputes to the wound not healing by the first intention.

"From this time (continues Sir Everard) the patient was not under my direction; but I understood that he tried the effect of large doses of opium, which did not afford relief. He was then induced to employ electricity, which was also unsuccessful; and he died in a fit, which at the time was believed to be apoplexy, about five months after the operation had been performed; but, as the body was not examined, the nature of the fit could not be ascertained.

"In this case, some of the branches of the median nerve had acquired from disease an unnatural power of contraction, which was made evident by the operation; and there is every reason to believe that the spasmodic attacks which took place were in reality convulsive motions in the nerves themselves, which excited corresponding contractions in those muscles which were under their influence."

Mr.

Mr. Pring, with much prudence, prefers resting his references on cases related by others who were unacquainted with his opinions. To this impression we are to impute the relation of the above cases, which, with all his abbreviations, occupy more room than appears to us necessary, as the result only shews what is pretty well known, our incapacity to ascertain any limited laws to the phenomena attendant on diseased nerves.

"We come now (says Mr. Pring) to speak of tetanus, the nature of which will be best described by a few examples. The following is a case of trismus, which occurred without being preceded by a wound.

"A woman, who had been standing in the street about an hour in an intensely cold day (in the winter of 1813-14), was seized on a sudden with a torpor and incapacity of the whole body: she was perfectly sensible, but she was unable either to move or to speak. She was taken into a house, and made warm by a fire; and in about an hour the motions of the limbs were restored.

"At this time I saw her, and found the jaw so closely locked that it appeared impossible to introduce a sixpence between her teeth. I directed the face and neck to be rubbed with a stimulating embrocation, and that an injection should be administered. The pulse was quite natural.*

"Two hours afterwards I saw her again; and, by using some force, was enabled to introduce the handle of a spoon between her teeth, and in this way, by a little mechanical violence, succeeded in opening her mouth so as to give her six grains of calomel and a cathartic draught. As soon as the spoon was withdrawn, the jaw became again immoveably fixed. I left her for the night, merely ordering the injections to be repeated every three or four hours.

"My reason for directing the treatment so specifically to the bowels was, that, as far as I could understand from those who lived with her, she had had no alvine evacuation for five or six days. In this supposed connexion between the state of the bowels (establishing perhaps the predisposition) and the symptoms I was not deceived.

"On the following morning, my patient was in the same state as on the preceding night. I was able, though not without much difficulty, to open her mouth by the same means, and to give her more cathartic medicine. This operated in about two hours afterwards, and produced very copious discharges from the bowels. In less than an hour after the first effect of the cathartics, she was able to open her mouth, and to talk intelligibly; though, before this effect,

"* Dr. Parry makes the following important remark on the pulse in tetanus: 'If, in an adult, the pulse, by the fourth or fifth day, does not reach 100, or perhaps 110, beats in a minute, I believe the patient almost always recovers. If, on the other hand, the pulse on the first day is 120 or more in a minute, few instances will, I apprehend, be found in which he will not die.' Cases of Tetanus and Rabies Contagiosa, p. 18."

not not the slightest abatement of the spasm had taken place. A little stiffness remained about the neck, which gradually left her.

"A waggoner abraded the skin on the back of the hand for about the extent of half an inch. The injury was superficial, *no tendon was exposed*, and the wound healed in about a week. A few days after, the man complained of stiffness of the muscles of the neck: the jaw was speedily locked, a state of rigid spasm affected the whole system of the voluntary muscles, and the complete state of tetanus was thus established, in less than 24 hours from the notice of his first symptoms. There was scarcely time for the trial of remedies, assistance not being immediately procured; some of those, however, which have been recommended (and they are all, perhaps, equally efficient) were enforced: and the man died in about 48 hours from the commencement of the spasm.

"A man's leg was grazed by a cart-wheel just above the ancle. The exposure was *superficial*, though the contusion was severe. The wound became sloughy; and the state of tetanus was rapidly established. In this case, wine and opium were given in large quantities; and blisters were applied on the back of the neck and between the shoulders. Finally, there was not a voluntary muscle in the whole body which was not affected with spasm, and the agony of the symptoms in their progress was intense. On the night of the third day from the commencement of the attack, it was directed that this man should be put into the warm bath: he died on the same night, very speedily after his removal from thence."

We have copied these cases to shew how exactly the disease follows the same course as in the days of Hippocrates and Celsus, which is confirmed by the extract from Dr. Parry. In our review on Dr. Parry's work (see London Med. and Phys. Journal, vol. xxxiii. page 317), we particularly remarked this coincidence in the cases related by all authors, ancient and modern, though none of the moderns, excepting Mr. Hunter and Dr. Parry, have attended to the law. We have reason to believe Mr. Pring's work was prepared for the press before this remark met his eye. We refer to the case of the woman, in which the effect of a purgative seems, by implication, to be held out as the means of the cure. But we are told that the pulse was quite natural, and that the spasm was confined to the fauces. It appears, however, by another passage, in which this case is alluded to, that all the opinions concerning the digestive organs as the universal cause of disease, are considered unintelligible or unphilosophical.

"It is much to be lamented, (says Mr. Pring, in a subsequent note,) that, notwithstanding the seeming progress of our art, and the multiplicity of detached remarks on the subject, we have never yet been presented with the faintest resemblance of a scientific view of the natural or pathological relations of the abdominal viscera."

We sincerely join in lamenting so much decision from such uncertain premises. In most cases, as in that we have just noticed, when the diseased action ceases, the organs become obedient to stimuli which produce no effect on them when under the influence of a more powerful cause. The following is the spirited manner in which the various remedies for this formidable disease are enumerated.

"Of the cases of tetanus, an immense number have been recorded: they furnish the materials of a copious compilation, which would serve only to exhibit a striking picture of the disease. In these histories, it would be perceived that no remedy which has been supported by the faintest analogy has been neglected: the event has been alike fatal under all. It is true, that the means which have failed in subsequent trials, have been said to succeed by those who proposed them: it must also be remembered, that the disease has sometimes terminated favourably where no efficient means have been enforced. I have heard of such cases; and one of trismus has fallen under my own notice, on which all the probable resources of the pharmacopœia were exhausted without effect. The case became chronic, and was abandoned in despair: eventually, the patient recovered, when all medical treatment had been for some time discontinued.

"The following is a partial list of the means which have been employed for the cure of tetanus. Bleeding, moderately and immoderately; blisters, sudorifics, the warm bath, the cold bath, and cold effusion: cold has also been applied to the body by bandages wetted with æther; opium in all doses,* camphor, musk to more than the amount of a hundred grains in a few hours, ammonia, assafœtida, acids, alkalis; mercury in inordinate quantities, rubbed into the surface and taken into the stomach, so as to produce salivation in 24 hours; wine, brandy, and electricity, have been used to stimulate; and no article in the list of narcotics has been omitted to diminish excitement.†

"The wound has been soothed by poultices and fomentations, and its surface has been destroyed by caustics. The limb has been

---

"* A scruple of solid opium has been given every hour, and a dram as a night-dose; opium, when given in this way, has been found in the stomach, after death, in a quantity sufficient to have killed the patient if he had recovered from the disease.

"† Mr. T. Duncan, of Grenada, has lately published a case of tetanus, which recovered under the use of tobacco injections: the pulse in this case never exceeded 94. (See note, p. 214.) The case alluded to is published in No. XLII. of the Edin. Med. and Surg. Journal."

This case furnishes another instance where a remedy had the credit of curing a disease which, according to the observations of the ancients, and of Mr. Hunter and Dr. Parry only among the moderns, would have ceased spontaneously.—EDIT.

suffered to remain, and the limb has been amputated. Patients, it is said, have been cured by all these means; they cannot be cured by any of them; and, finally, they sometimes recover without any treatment at all. We must conclude that the worst forms of tetanus are not curable by any known remedy; and that, of the intermediate ones, those appear to recover under every treatment, which would otherwise get well without the interference of art.

"The treatment of tetanus by cold immersion, laudanum, and wine, as introduced by Dr. Currie, of Liverpool, is that which is generally considered the best. The immersion in cold water has been said, when it fails to cure the disease, to produce an abatement of the severity of the spasms. Other cases are reported, in which the cold immersion has been prejudicial, increasing rather than relaxing the rigidity of the muscles.*

"The forms of tetanus are enumerated under the titles of trismus, emposthotonos, episthotonos, pleurosthotonos, &c.; for my own part, I consider these as no better than medical witticisms, and should be as well contented to acknowledge my ignorance in my own language."

After these remarks on the general character and tendency of tetanus, Mr. Pring enters into the inquiry how far our knowledge extends in the more minute pathology of the disease. He conceives that the opinion concerning a wounded tendon as a cause, is, to say the least, unfounded. From his own observation, he has found that the wound of a nerve has in some way preceded such an event; and the frequency with which the tendo Achillis, with many others, is ruptured or divided without such a consequence, furnish strong arguments in his favour. As a physiological argument, too, the author found, by experiment, that, when the trunk of an artery supplying a large muscle is divided, the contraction of that muscle from any external stimulus is very trifling, and of short continuance. Hence it is presumed, that nothing but some affection of the nerve could induce those powerful and long-continued contractions which characterise this formidable disease. The next consideration, therefore, is the nature of the change in the nerve which induces such an effect. That it is not inflammation, is satisfactorily proved.

"It has been frequently glanced at (says Mr. Pring) in the preceding pages, and demonstrated in that part of the subject of inflammation which treats of the relation of proximity, &c. that the nerves are furnished with latent properties; or else, that the properties which are displayed in their phenomena, are liable to be mo-

"* Vid. Cases of Tetanus and Rabies Contagiosa, p. 4, by Dr. Parry."

dified, so as scarcely to be recognised by any analogy, under the influence of some of the numerous causes which affect them.

"These latent properties obtain variously in the parts of the system; and they are the agents of a relation which has been termed sympathetic.* They have been shewn to be of two kinds, natural and constant, and acquired or temporary. A relation of the former kind has been shown to exist in the sciatic nerve at a distance of half an inch from the spine; and an example of the latter would be found in the remote parts of the same nerve, provided such parts should, as an anomaly, display the same phenomena as those which result from the application of the same cause at a point which is contiguous with the medulla spinalis. Examples, bearing some analogy to both of these, are almost infinite in an animal body. The connexion between the parts which are thus circumstanced has been called a specific relation, and the changes which are induced by external causes, specific effects; and we must continue to use this language until we are informed of the agents involved, or have reduced them to a similitude with some more familiar acquaintance."

Pleased as we are with our author's boldness in discarding undefined terms, we should, in any other writer, be induced to consider the note to this passage as captious. "Sympathy" means at least as much as "specific relation," and is as convenient a term to express an effect. The swelling of the testicle is a disease, and the stricture or inflammation of the urethra is a disease also: *ergo*, there is a συμπασχω, or suffering together, or "suffering with." Now, where is the mutual suffering between the cannon-shot and the man's head? In the latter case the agents are pretty well known, or may be guessed. In sympathy, we pretend to no knowledge of agents, but, having detected certain laws, we are entitled to give to the result a name as expressive as we can discover. Another material difference between the allusions is, that, as the author remarks afterwards, "in many examples of sympathy, the secondary effects ceases with the removal of the original cause." But this can never happen by any restoration of the cannon-ball to its original place. If it is only meant that there is not so constant an uniformity in sympathetic actions as to admit the term without any re-

"* The word sympathy, as implied by its derivation (συμπασχω, to suffer with), is applied only to denote *effects*. It is meant to express connected occurrence; as a testicle is inflamed by sympathy with the urethra, or, in other words, there are strictures in the urethra, and the testicle swells: the word is not indicative of a *process*, or a *mode*: and it is used in medicine as it may be used in the case of a man who is killed by a cannon-shot, namely, that the man sympathized with the cannon; the agents not being glanced at in either instance."

striction, striction, we are then still further from the cannon-ball allusion. Sympathy is only as regular as most of the actions we can detect in the living body, subject, as they all are, to be modified by climate, age, sex, and various idiosyncrasies.

We shall here cease our animadversions on this valuable little tract, and could almost have wished that the author had stopped, or had considerably curtailed the little that remains. It is among the numerous proofs which are occurring to us how easy it is to shake the fabric of a theory, and how difficult to form a new one. We cannot, however, conclude without our sincere thanks for the information we have received, and our earnest entreaties that Mr. Pring will continue his experiments. From the pleasantry with which he has enlivened his subject, we take the liberty of advising, that, in a future edition, or in the future relation of his experiments, more familiar terms may be adopted. *Neurilema* seems to us unnecessary—*Centres of nerves* not less so; or, if the author finds them convenient, the work should begin with an explanation of terms. With much candour he expresses a readiness to "acknowledge his ignorance in his own language:" we only wish him not to obscure what he has really discovered, and what requires no embellishment to enhance its value.

---

*Suggestions for the Relief of the Sick Poor, and the Improvement of the Medical Profession, in Great Britain.* By John Dunn, M. R. C. S. Surgeon, Pickering, Yorkshire. 8vo. pp. 24. 1817.

This little tract, which appears without a publisher's name, contains suggestions which arose in the author's mind from the laudable and necessary attempts of the Worcestershire Society.

England is perpetually boasting of its numerous charities. We are ready enough to admit their number; but either we have more necessity for them than other nations, or all, except ourselves, must be in a dreadful condition. Parish-officers are proverbially either indiscriminately generous, or severely churlish. Can we wonder at this, when we know that the office of overseer is, for the most part, refused by all the better-informed and better-conditioned, and undertaken for hire by the needy and unfeeling? Where are now the Ladies Bountiful, who used to administer to all their suffering neighbours?—Where the rich lord of the manor, who conceived it his duty to protect all his industrious tenants?

—Where

—Where the commissioners of the peace who, from their mansions, thought it their duty to reconcile ignorant and quarrelsome neighbours, and, at quarterly sessions, to learn the distresses of the county, the causes of them, and to apportion relief accordingly?—Where the resident clergyman, who conceives that the poorest of his flock has the greatest necessity for his advice, not only in the pulpit and on Sundays, but at all times, and particularly in times of need?

It will not be suspected, that we conceive all these characters extinct.—Far otherwise: but, wherever they still exist, the poor are rendered comparatively comfortable, the merits and labours of the medical man are duly appreciated, his rank and talents respected, and, if he fails to grow rich, he receives that kind of estimation which sweetens his labours and encourages his perseverance.

The proposal of Mr. Dunn is, in many respects, judicious. We shall give it in as few words as possible, that it may furnish hints whenever the subject meets with due consideration.

"The division of the kingdom into districts, and the appointment of respectable professional men, with a fixed salary, to furnish medicines, and attend every case certified by the overseer of the parish in which the sick person resides.—The allowance of £100 per annum, for a division of about 500 paupers, I think would be deemed an adequate remuneration."

The population of England in the year 1801, amounted to 10,942,616.

"The number of poor relieved in and out of workhouses, amounted to 1,039,716, which, deducted from the resident population, 8,872,980, will make a proportion of about twelve in a hundred. Taking it for an eighth, now that the distresses of the country are increased, a population will remain of above seven millions capable of paying the necessary expences.

"A million paupers, at the rate of five hundred for each apothecary, would require 2000 medical persons to attend them; who, being allowed a salary of £100 each per annum, would cost the country £200,000, which, with a population of seven millions, would just amount to sixpence farthing each, exclusive of those who are receiving parish relief.

"According to the calculations of Davenant and Brakenridge, the number of persons in a house in England and Wales, amounted to six; of Mr. King, only four and a half; Dr. Price thought five too many; but the fact now appears to be, five and three-fifths. Say, for convenience, six, which, multiplied by sixpence farthing, will make three shillings and three halfpence to each family, per annum; a sum, if collected monthly or quarterly from the wages of the labour-class, would never be felt; and, from trades-people and the higher orders, would almost amount to nothing.

"A capitation-

"A capitation-tax is generally esteemed improper, as it falls more severely upon the poor; but a tax intended solely for their own interest, and of such trifling amount, could not be considered in any other view than as a fair price for benefits received. The difficulty and trouble attending this mode of taxation, is a more forcible objection, and, if it should be deemed inconvenient, a species of income-tax might be raised, which would operate in the mildest manner.

"From the returns of 1804, the income-tax of the nation, at one shilling in the pound, produced £4,650,000: a penny in the pound would, therefore, raise £387,500,—a sum more than adequate to the end proposed."

In an Appendix, the author very judiciously proposes, that, as the poor-rate is a kind of discretionary tax, the sum for remunerating the surgeon might be collected at the same time.

To facilitate the labours of the surgeon, Mr. D. proposes, that he should be expected to instruct a certain number of females to attend as midwives on ordinary occasions, who should receive five shillings for each delivery.

This tract is very judiciously compiled, and should be reserved as a reference by all who attend to the state of the poor, whether in sickness or health.

---

